# Germanium-on-Silicon Waveguide-Integrated Photodiode with Dual Optical Inputs for Datacenter Applications

**DOI:** 10.3390/mi17030386

**Published:** 2026-03-23

**Authors:** Itamar-Mano Priel, Shai Cohen, Liron Gantz, Yael Nemirovsky

**Affiliations:** 1 Electrical and Computer Engineering Department, Technion-Israel Institute of Technology, Haifa 320003, Israel; 2 Nvidia, Yokneam 2069200, Israel; cohens@nvidia.com (S.C.); lironga@nvidia.com (L.G.)

**Keywords:** silicon photonics, waveguide-integrated photodiode, high power optical interconnect, dual optical inputs, electro-optical bandwidth, space-charge effect, datacenter optical interconnects, germanium-on-silicon photodetector

## Abstract

As the exponential growth in advanced compute workloads drives intra-datacenter interconnects to ever increasing bitrates, optical networking equipment has risen to the challenge by shifting from NRZ signaling to bandwidth efficient modulation methods such as PAM4. As these modulation schemes introduce an inherent SNR penalty, maintaining low bit error rates (BER) forces optical links to operate at significantly higher optical powers. However, increasing the optical power leads to photodetectors reaching one of their fundamental bottlenecks caused by the space-charge effect, limiting their ability to provide a high-speed response under high-power illumination. This work presents the design, fabrication, and characterization of a waveguide-integrated photodiode with dual optical inputs (DIPD) designed to overcome this limitation. Specifically, we demonstrate that combining a dual-fed architecture with targeted cross-sectional geometric optimizations effectively distributes the photocurrent density to delay the onset of space-charge saturation. Experimental validation demonstrates a high responsivity of ≈0.91 [A/W] (for O-band wavelengths) and a large electro-optic bandwidth (EOBW) of ≈58 [GHz], all under high-power illumination and CMOS driving voltages.

## 1. Introduction

Silicon photonics has emerged as a promising technology platform for high-speed communications systems [1], particularly in datacenter applications. The ability to manufacture and interconnect photonic and electronic integrated circuits using CMOS-compatible fabrication processes has opened avenues for fast, energy-efficient, and cost-effective optical interconnect over distances and bitrates unattainable with conventional copper interconnects [2]. As industry progresses toward terabit-scale communication, the performance requirements for fundamental electro-optic components such as modulators and photodetectors have intensified. To support higher modulation formats (such as PAM4), photodetectors must operate at increasingly high optical power levels while maintaining or even increasing their EOBW in order to preserve low bit error rates across the optical link [3]. However, scaling traditional silicon photonics photodetectors to high speeds and high powers presents a fundamental physical challenge: the space-charge effect [4], which stems from a combination of high optical power and the classic exponential decay absorption profile of light (often sharp due to the strong absorption coefficient of traditionally employed germanium(, leading to high local densities of photogenerated charge carriers. As the charge carriers begin to drift towards the electrical contacts of the photodiode, they create a self-induced electric field, which opposes the internal drift field, effectively screening it. Consequently, the velocities of charge carriers are drastically reduced, and the EOBW is severely constrained [5].

In this work, we present a dual-pronged approach for mitigating the negative effects of space-charge: by designing the DIPD’s cross-section for a longer 1/e absorption length and the dual-input architecture, we effectively distribute the optical load across the photodetector and reduce otherwise high local concentration of photogenerated charge carriers, maintaining high responsivity and EOBW. While dual-input designs have been previously explored [6,7], our work demonstrates that synergizing this architecture with active cross-sectional absorption optimization yields further EOBW improvements under high power without increasing leakage current or sacrificing responsivity.

## 2. Design and Geometry

**Figure 1:**
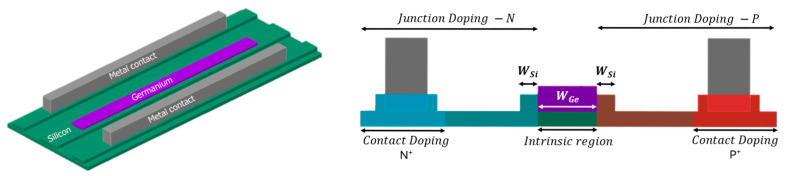
DIPD structure. (**Right**)—Cross-section with PN doping mask regions indicated by blue and red overlays. (**Left**)—Isometric view: the absorbing Ge (purple) is embedded within the Si (green) rib waveguide, with metal contacts (grey).

The DIPD structure features a lateral PIN junction in a silicon rib waveguide fabricated on a standard silicon-on-insulator substrate [8], with an absorbing germanium (Ge) layer grown in an etched recess. This approach was chosen for its high performance and CMOS-foundry compatibility: while all-silicon photodetectors based on bulk-defect absorption exist and have been experimentally demonstrated, they suffer from very weak absorption coefficients, requiring non-CMOS-compatible bias voltages [9,10,11] extremely long devices (hundreds of microns) [10], which add parasitic capacitance and have a significant size footprint, or rely on resonant structures for absorption enhancement [9], which require thermal tuning and are spectral bandwidth limited. In contrast, Ge, with a high absorption coefficient at both C- and O-band wavelengths, is available in commercial SiPh platforms, making it a “go-to” choice for photodetector designs. To demonstrate and study the space-charge effect, we simulate and design a Ge-on-Si waveguide-integrated photodiode cross-section based on a commercial foundry’s silicon photonics platform. 3D finite difference time domain (FDTD) simulations were performed using perfectly matched layer (PML) open boundary conditions with a TE polarized mode source. Standard material models (Palik) were utilized for the complex refractive indices of silicon and germanium at the O-band target wavelength of 1310 nm. The baseline geometry was intentionally optimized for strong absorption. The DIPD’s geometry is shown in Figure 1.

**Figure 2:**
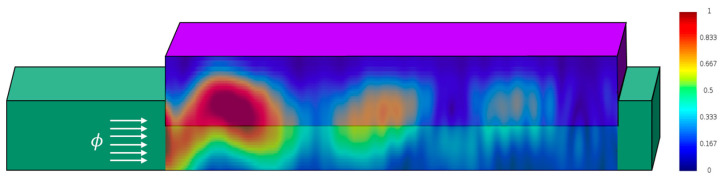
FDTD simulation of the normalized optical power distribution at a wavelength of 1310 nm along a longitudinal cross-section of the propagation axis (single-input illumination, with incident optical flux ϕ entering in the direction of the arrows). The simulation demonstrates the localized high-intensity regions at the device where absorption and photogeneration is strongest, driving the space-charge effect.

Finite differences time domain (FDTD) simulations illustrated in Figure 2 indicate that the baseline DIPD cross-section design with Ge width of *W_Ge_* = 0.75 [μm] and silicon rib width of *W_Si_* = 0.15 [μm] is expected to achieve close to maximum theoretical responsivity at a length of 10 [μm], with a 1/e absorption length of approximately ≈1.4 [μm].

## 3. Methods and Results

**Figure 3:**
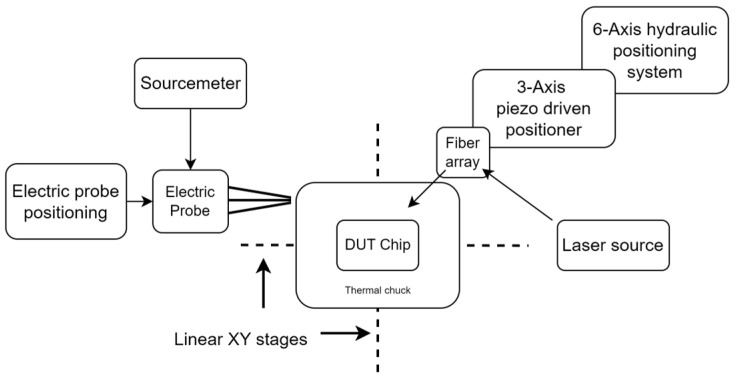
Block diagram of the experimental characterization setup. The system integrates precise optical alignment stages, a tunable laser source, and a sourcemeter for electrical biasing and readout of the waveguide-integrated photodiode.

Measurements were carried out on a temperature-stabilized die-level tester, based on Physik Instrumente’s F712 Fast alignment positioning for coupling light into the chip, utilizing surface grating couplers, optimized for TE polarization. Photocurrent and responsivity measurements were performed via a semiconductor laser (Santec TSL570) and a precision source-measure unit (Keithley 2400), configured as shown in Figure 3.

**Figure 4:**
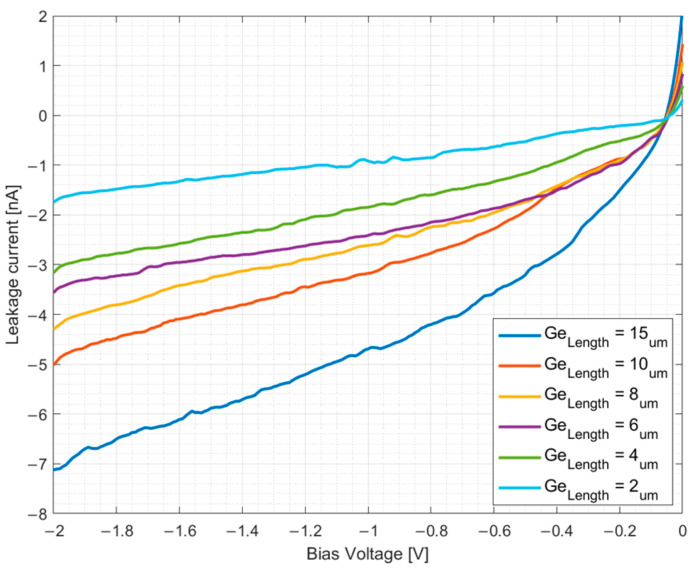
Measured leakage current versus reverse bias voltage for DIPD structures with varying germanium lengths (*L_Ge_*). The results show the I–V characteristics for device lengths ranging from 2μm to 15 μm, demonstrating low dark currents below 8 nA at −2 [V].

Leakage current was measured with precision I–V sweeps, and the results are presented in Figure 4.

To validate FDTD simulations, the cutback method was employed, in which DIPDs featuring the same cross-sectional dimensions with increasing lengths were fabricated. Responsivity characterization is based on fitting the measured photocurrent to the following equation:

(1) $$I_{\lambda }=R_{esponsivity}\cdot P_{in-PD}+I_{leakage}$$

**Figure 5:**
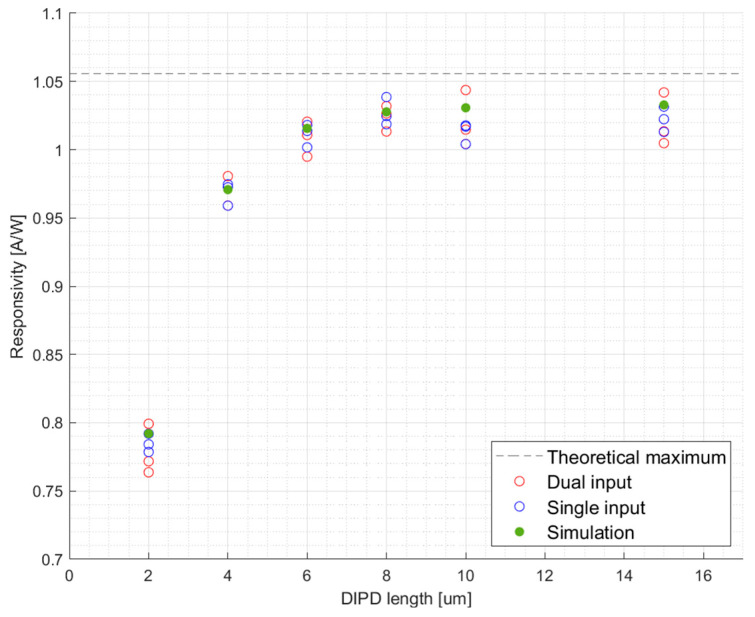
Responsivity vs. DIPD’s Ge length measurements from 3 dies compared to FDTD simulations. FDTD simulations yielded identical responsivity for both single and dual input configurations; therefore, a single simulated dataset is plotted.

The responsivity results obtained from three separate dies for both dual and single input illumination schemes are presented in Figure 5.

We observe a strong correspondence between responsivity predictions from FDTD simulations and the measured responsivity values. The observed discrepancies are primarily attributed to manufacturing tolerances.

Using the strong agreement between the simulated absorption profiles and measured responsivities demonstrated in Figure 4, we extract a 1/e absorption length of approximately ≈1.4 [μm], confirming that nearly all optical power is absorbed in the first few microns of the device (*L_eff_* ≈ 4 [μm], resulting in a highly localized volume of charge carriers. Using these results and the analytic derivations in Appendix B, we estimate a threshold carrier concentration and photocurrent at which our baseline device transitions to a space-charge limited (SCL) regime. We assume a simplified uniform charge carrier distribution, serving as a lower bound estimation for local carrier density; since the actual absorption profile is exponential, peak densities at the facet of the DIPD are significantly higher. The critical carrier density ($N_{SCL})$ required to screen the internal drift field at a bias voltage of *V_bias_* = −2 [V] is given by

(2) $$\frac{N_{SCL}=2\varepsilon_{0}\varepsilon_{r}|V_{bias}|}{qW_{Ge}^{2}}$$

We map this threshold carrier density to a threshold photocurrent generated by the active volume of the device *V_active_* via carrier transit time $\left(\tau_{t}r=\frac{W_{Ge}}{v_{sat}}\right)$ using

(3) $$I_{threshold}=\frac{Q_{total}}{\tau_{tr}}=\frac{q\cdot N_{SCL}\cdot V_{active}}{\tau_{tr}}$$

Substituting the expression for $N_{SCL}$, we calculate the threshold photocurrent:

(4) $$I_{threshold}=\frac{Q_{total}}{\tau_{tr}}=\frac{2\varepsilon_{0}\varepsilon_{r}|V_{bias}|\cdot v_{sat}\cdot L_{eff}\cdot H_{Ge}}{W_{Ge}^{2}}\approx 60\;[\mu A]$$

While this threshold represents a theoretical onset of field screening rather than a total link failure, it indicates that, at standard operating currents of 500~1000 [μA], the DIPD would operate deeply within the space-charge limited regime.

## 4. Electro-Optic Small Signal RF Characterization

**Figure 6:**
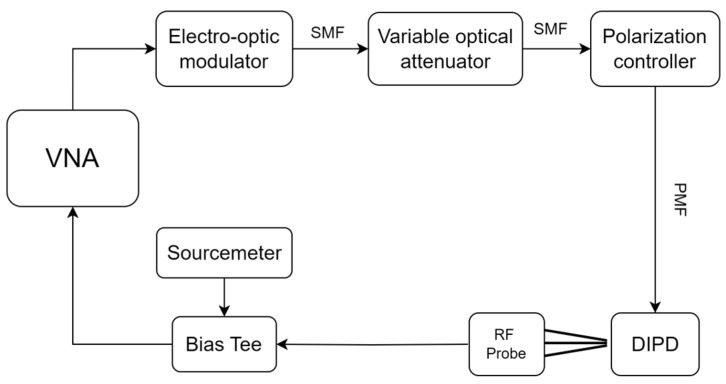
Schematic of the RF characterization setup. The setup measures the S21 frequency response by modulating the optical input via a VNA-driven modulator. The resulting photocurrent from the DIPD is extracted via a bias tee and measured by the VNA.

The electro-optic small signal S21 response (EOS21) was measured using the same die-level tester. The measurement setup, illustrated in Figure 6, utilizes a vector network analyzer (VNA, Rohde Schwarz ZNA67) along with a calibrated EO converter with an internal CW laser source (Thorlabs MX70G). Modulated optical output from the modulator was passed through a variable optical attenuator (VOA, Keysight N7762A) and a polarization controller (General Photonics PSY201). The modulated illumination was directed to the DIPD, and the resulting modulated photocurrent was then collected at the VNA’s electrical port via RF probe and bias tee.

**Figure 7:**
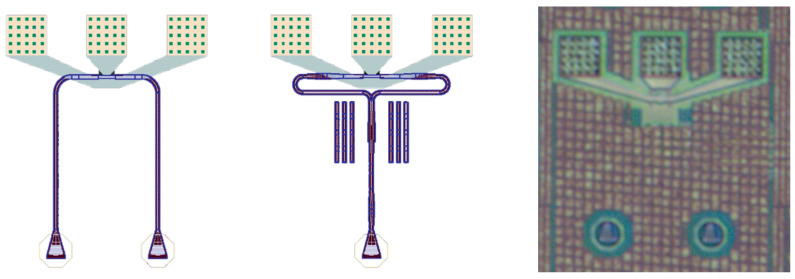
Top-down layout schematics and optical microscope image of the fabricated test structures. (**Left**) Layout of a dual-input photodiode test structure fed by independent grating couplers. (**Center**) Layout of the primary dual-input architecture, where a single grating coupler feeds an on-chip 50:50 optical splitter with balanced paths leading to the photodiode. These passive optical routing elements consist of the foundry’s standard single-mode silicon rib waveguides and grating couplers. Both layout schematics detail the optical routing and the ground–signal–ground (GSG) RF contact pads. (**Right**) Optical microscope image of the fabricated electrodes, showing the high-speed GSG RF contact electrodes.

RF measurements were carried out via ground–signal–ground (GSG) probing, and the system was de-embedded to the probe tips to isolate the device’s true S21 response, as demonstrated in Figure 7.

**Figure 8:**
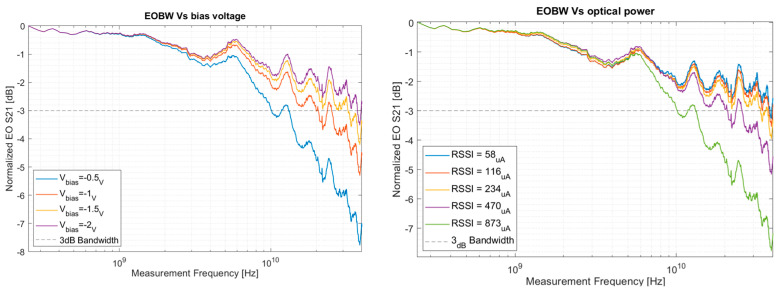
Normalized EOS21 measurements. (**Left**) increasing bias voltages, all at a constant average optical power correlating to received signal strength indicator (RSSI) of approximately $\approx 800\left[\mu A\right].$ (**Right**) measurements with increasing RSSI, at a constant bias voltage of $V_{bias}=-0.5\;[V]$.

Single optical input RF measurements are demonstrated in Figure 8.

In these measurements, we report performance against the generated DC photocurrent (RSSI) rather than external optical power to isolate intrinsic device performance from variable fiber-to-chip coupling losses, directly correlating our measurements to the internal charge density driving the space-charge effect. Consequently, we observe a direct correlation between EOBW and bias voltage and an inverse correlation with average incident illumination power. The positive relationship with bias voltage is anticipated, as the increased bias reduces the transit times of photogenerated charge carriers by stronger electric drift field. The observed decline in EOBW with higher RSSI demonstrates the bandwidth degradation caused by the space-charge effect.

To overcome this limitation, we implemented a design optimization strategy focused on two physical mechanisms: reducing the carrier transit time and lowering the local photocurrent density. First, the transit time was reduced by narrowing $W_{Ge}$, which directly improves EOBW by reducing the carrier drift duration. Second, to prevent high local charge densities, we distributed the optical load using two methods: widening the Si rib to increase the 1/e absorption length and implementing a dual optical input architecture fed by balanced 50:50 optical splitters. These combined techniques effectively distribute photogeneration alongside the device and balance the optical power.

To validate this approach, optimized DIPD cross-sections featuring reduced Ge widths ($W_{Ge}=0.5\mu m,\;0.35\mu m$), increased Si rib width ($W_{si}=0.6\mu m$) and an increased Ge length $\left(L_{Ge}=20\;\mu m\right)$ for compensation of the expected absorption reduction were fabricated along with a copy of the baseline design.

**Figure 9:**
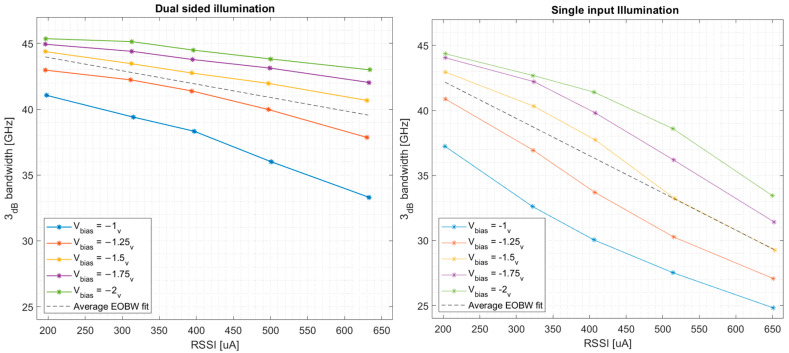
EOBW vs. RSSI for the baseline DIPD cross-section design ($W_{Ge}=0.75\;[\mu m]$). (**Left**) Dual-input illumination achieved via passing the modulated light through an on-chip 50:50 optical splitter, with balanced optical paths leading to the DIPD’s optical inputs. (**Right**) Single-input illumination scheme. The space-charge’s EOBW degrading effect is most obvious at lower bias voltages and higher RSSI values. Furthermore, it is apparent that dual-input illumination extends the EOBW to higher frequencies.

In order to better analyze measurement results, we plot the EOBW vs. RSSI for our baseline design in Figure 9.

**Figure 10:**
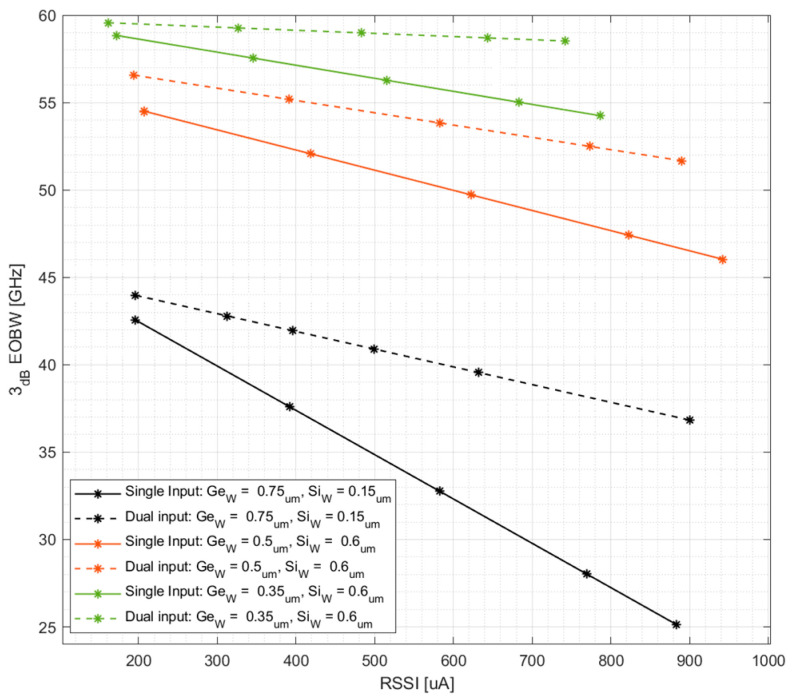
Performance comparison of the 3 dB electro-optical bandwidth (EOBW) versus RSSI for the baseline $\left(W_{Ge}=0.75\;\left[\mu m\right]\right)$ and optimized $\left(W_{Ge}=0.5\;\left[\mu m\right]\;and\;0.35\;\left[\mu m\right]\right)$ DIPD cross-sections. Solid lines represent single-input illumination, while dashed lines correspond to the dual-input configuration. The results demonstrate two optimization mechanisms: reducing the germanium width increases the intrinsic bandwidth by minimizing carrier transit time, while the dual-input architecture, combined with cross section optimizations mitigate the space-charge effect, resulting in significantly higher sustained bandwidths at high optical powers compared to single-input operation.

For methodical comparison between the relative performance of different DIPD designs, EOBW is averaged at each RSSI point over all bias voltages, and the resulting data points are fitted to a linear curve. With a single curve representing the performance merit of each DIPD design, we are now able to present all designs collectively in a single plot, demonstrated in Figure 10. Thus, while Figure 9 demonstrates the performance of a single DIPD cross-section, Figure 10 compares the performance across designs.

For the reader’s reference, Table 1 presents a comparative overview of this work alongside related devices, incorporating reported performance metrics from recent years. It is worth noting that, while metrics such as noise equivalent power (NEP) and specific detectivity ($D^{*}$) are standard for evaluating free-space and imaging photodetectors, they are fundamentally less applicable to waveguide-integrated datacom photodiodes, as in PAM4 datacenter links; the system is not limited by the absolute minimum detectable signal at the noise floor but rather by bandwidth degradation and saturation under high-power illumination. Furthermore, normalizing performance by physical area (as in $D^{*}$) loses its physical relevance when light is confined within a single-mode guided volume rather than captured from a free-space aperture. Consequently, the standard figures of merit in this domain and the focus of our comparative analysis are responsivity, electro-optic bandwidth (EOBW), and dark current evaluated against specific received signal strength indicator (RSSI) levels, as these directly dictate the optical link budget and high-power operational limits.

## 5. Summary, Conclusions, and Future Work

This paper presents the design, fabrication and characterization of a high-speed, dual-input waveguide-integrated photodiode (DIPD) tailored for high-power optical communication systems in datacenters.

Experimental results demonstrate that the device achieves a high responsivity of 0.91 [A/W] and a large EOBW of ≈58 [GHz] while maintaining a low dark current of ≈70 nA at −1.5 V bias. A comprehensive comparison with state-of-the-art devices is provided in the Figure of Merit table in Appendix A. Significantly, this work provides a clear experimental identification of the space-charge effect and demonstrates a successful mitigation strategy. By optimizing the cross-sectional geometry to distribute absorption and utilizing a dual-input architecture for optical load balancing, the DIPD effectively overcomes carrier screening limitations to enable high-speed operation at elevated optical powers.

While this work focused on optimizations for lateral cross-sectional dimensions ($W_{Ge}$ and $W_{Si}$), future generations of high-power waveguide photodiodes could benefit from additional co-optimization of the vertical along with the horizontal layer parameters. Specifically, employing a thinner germanium layer could further distribute absorption along the propagation axis, delaying the onset of the space-charge effect. However, this degree of freedom must be carefully co-designed with the lateral waveguide dimensions to prevent the optical mode from delocalizing into the surrounding silicon waveguide. Excessive delocalization would severely reduce the optical confinement factor within the active region, effectively decoupling the light from the germanium and completely halting absorption. Therefore, striking the right balance across all three dimensions through careful co-design will be the key to unlocking the next tier of power handling in terabit-scale optical links.

## Figures and Tables

**Table 1:** Comparison of this work with previously reported devices and their performance metrics.

Ref.	Device Type	Photodetector Architecture	Responsivity [A/W]	Quantum Efficiency [%]	EOBW [GHz]	Bias Voltage [Volt]	RSSI $\left[\mu A\right]$	Leakage Current$\left[\mu A\right]$
[12]	Ge-on-Si waveguide PD	Single input	0.8	80	N.A.	N.A	N.A.	N.A.
[13]	Ge fin in Si waveguide PD	Single input	0.3	24	265	2	350	0.2
[14]	Phonon-assisted absorption in Si micro-ring	Single input	0.35	36.8	14	2	20	0
[15]	Ge-on-Si waveguide PD with inductive peaking contacts	Single input	1.09	87.2	42.5	4	35.8	3.5
[16]	Ge-on-Si waveguide PD	Single input	0.7	56	17	6	N.A.	0.07
[10]	Bulk defect absorption in Si waveguide	Single input	0.175	1.4	N.A.	5	N.A.	$5\cdot 10^{-4}$
[9]	Bulk defect absorption embedded in racetrack resonator	Single input	0.14	11.2	N.A.	10	N.A.	0.0002
[11]	Bulk defect absorption embedded in Si micro-ring	Single input	0.15	15	N.A.	13	N.A.	0.04
[17]	Traveling wave photodetector	Multiple input	0.76	60.8	31.6	3	2000	4
[6]	Side-coupled high-power Ge on Si photodetector	Dual input	0.8	76	53	3	5300	0.031
[7]	Large bandwidth high-power Ge on Si photodetector	Dual input	0.8	76	48	3	2100	0.007
This work	Dual-input waveguide photodiode	Dual input	0.91	86.6	58	1.5	920	0.07

## Data Availability

Data is available on reasonable request from the correspondence author.

## Abbreviations

The following abbreviations are used in this manuscript, in order of appearance:

NRZ	Non Return to Zero
PAM	Pulse Amplitude Modulation
SNR	Signal to Noise Ratio
BER	Bit Error Rate
DIPD	Dual-Input Photodiode
EOBW	Electro-Optical Bandwidth
CMOS	Complementary Metal-Oxide-Semiconductor
Ge	Germanium
FDTD	Finite Differences Time Domain
PD	Photodiode
SCL	Space-Charge Limit
EOS21	Electro-Optic Small Signal S21 Response
VNA	Vector Network Analyzer
SMF	Single-Mode Fibers
PMF	Polarization-Maintaining Fibers
VOA	Variable Optical Attenuator
RF	Radio Frequency
RSSI	Received Signal Strength Indicator

## Appendix A

DIPD performance is shown in Table A1.

**Table A1:** DIPD performance.

$W_{\mathrm{Si}}\;[\mu m]$	W_Ge_ [μm]	Leakage Current [nA] $V_{bias}=-2\;\left[V\right]$	Responsivity [A/W] $V_{bias}=-2\;\left[V\right]$	Average Single-Input EOBW [GHz] RSSI≈900 [μA]	Average Dual-Input EOBW [GHz] RSSI≈900 [μA]
0.15	0.75	3.07	1.02	26	36
0.6	0.5	10.56	1.00	46	52
0.6	0.35	24.09	0.91	54	58

## Appendix B

Space-charge threshold derivations begin with Poisson’s equation for a one-dimensional junction, relating the electric field gradient $\left(\frac{dE}{dx}\right)$ to the net charge density $\left(\rho \right)$:

$$\frac{dE}{dx}=\frac{\rho }{\varepsilon }=\frac{qN}{\varepsilon_{o}\varepsilon_{r}}$$

where *N* is the photogenerated carrier density, *q* is the elementary charge, and $\varepsilon_{r}\varepsilon_{0}$ is the permittivity of the semiconductor. Because the highly doped P and N contacts create a strong, laterally confined drift field that drops almost entirely across the intrinsic $W_{Ge}$, a one-dimensional approximation serves as a reasonable first-order model for estimating the primary carrier sweep-out dynamics and the onset of electric field screening. In the space-charge limited (SCL) regime, the high density of carriers ($N_{SCL}$) screens the external bias, causing the internal electric field to diminish. Following the physical model described by Williams et al. [3], we approximate the field distribution at this limit as a triangular profile dropping from a maximum $\left(E_{max}\right)$ to zero across the intrinsic width $\left(W_{Ge}\right)$. The applied bias voltage $\left(V_{bias}\right)$ is the integral of this field:

$$\left|V_{bias}\right|=\int_{0}^{W_{Ge}}E\left(x\right)dx\approx \frac{1}{2}E_{max}W_{Ge}\to E_{max}=\frac{2\left|V_{bias}\right|}{W_{Ge}}\;$$

Equating the geometric slope of this field $\left(\frac{E_{max}}{W_{Ge}}\right)$ to the physical gradient given by Poisson’s equation yields the critical density:

$$\frac{dE}{dx}\approx \frac{E_{max}}{W_{Ge}}=\frac{2\left|V_{bias}\right|}{W_{Ge}^{2}}$$

$$\frac{qN_{SCL}}{\varepsilon_{o}\varepsilon_{r}}=\frac{2\left|V_{bias}\right|}{W_{Ge}^{2}}\to N_{SCL}=\frac{2\varepsilon_{o}\varepsilon_{r}\left|V_{bias}\right|}{qW_{Ge}^{2}}$$

Using Ge-specific values,

$$\varepsilon_{r}\varepsilon_{o}=16\times 8.854\times 10^{-14}\left[\frac{F}{cm}\right],\;\;v_{sat_{Ge}}=v_{sat_{Ge}-holes}\approx 6\times 10^{6}\;\left[\frac{cm}{s}\right]$$

Specifying $V_{bias}=2\;\left[V\right]$ yields the theoretical carrier density at which electric field screening onsets:

$$N_{SCL}\approx 6.3\times 10^{15}\;[cm^{-3}]$$
